# International Congress of Gynæcology and Obstetrics

**Published:** 1896-06

**Authors:** 


					INTERNATIONAL CONGRESS OF GYNECOLOGY AND OBSTETRICS.
It has been determined to hold the second session of the Congress at
Geneva during the first week in September. Dr. Leith Napier, of 67
Grosvenor Street, London, W., is the Secretary for Great Britain.

				

